# Exposed Pulps

**Published:** 1884-09

**Authors:** J. D. Patterson

**Affiliations:** Kansas City, Mo.


### EXPOSED PULPS.

BY J. D. PATTERSON, D. D. S., KANSAS CITY, MO.

READ AT THE JOINT MEETING OF THE KANSAS AND NEBRASKA ASSOCIATIONS, MAY 4TH, 1884.

What to do with the exposed pulp has long been one of the chief questions to the dentist. The mode of treatment has undergone many changes. The correct method promulgated one year has been cast aside the year following, and while the advance guard has tested and discarded many plans, others are still using the old treatment that has been found worse than useless, showing that no one method has been found worthy of exclusive employment by all operators.

There are many barriers in the way of obtaining correct statistics of pulp capping. The cases upon which judgment chiefly rests are those where so much trouble has subsequently arisen that surgical interference has been found necessary. The exact condition of those that, after a certain time, do not cause uneasiness and are called successful, we do not surely know, for we are not justified in disturbing them, nor are we positive as to how long they will remain useful and comfortable. The pulp may be mummified, ossified, shriveled, or in nearly a normal state, so that it is difficult to obtain statistics that are not fallible. However, we can say that success in capping must depend upon circumstances of health, temperament, constitution, and especially upon local features of the exposure, which may be varied without limit. Every case demands individual consideration; and prescribed plans, which are often glibly laid down for absolute preservation of the whole class of exposed pulps, are extremely liable to prove unsatisfactory. The outcome shows this: Take two cases; alike, so far as a common observation extends, give them the same treatment, and the one will prove a success, the other a failure. The probable reason is not found in different manipulation, but because each case had different possibilities at the time treatment was given. We cannot apply an unerring rule to all cases, but may give the results of study and experience. After a pulp has undergone considerable irritation, inflammation and partial suppuration in the usual course of decay any attempt at pulp protection will be followed, not by the pulp regaining or continuing its functions, but by its obliteration as a pulp and its final entire destruction. Pain and inflammation may have been present to a considerable degree, and yet no doubt the pulp may be saved; but if the destruction of some portion of pulp tissue has occurred, the chances for successful capping are gone. Of course capping may be done with seeming success, but the increased vascular action which is excited for protection of pulp substance seldom stops short of pulp ossification, exostosis, and its following ills, if the pulp is not soon destroyed by suppuration. We are often pleased to term this ossification a secondary dentine, or osteo-dentine, when it is a heteromorphous mass of bone formation, which destroys the pulp and its functions. We find this condition often present when pulp treatment has been delayed until successive seasons of pain and inflammation have occurred, when if we endeavor to extirpate, we find it a difficult matter, as the entrance to the pulp chamber is blocked with nodules of this deposit. If the process stopped when a protection was afforded it would be well, but it does not. The accretion gains until the whole chamber is invaded, or a greater part of it. Very much has been said about saving a portion of a pulp, even if the major part of it is lost. The excising of these organs and the protection of the pulp in one fang of a tooth with two or three roots, when in the other fangs it is entirely removed, is spoken of by some as a common operation in their practice, and a sure success. No doubt these operations have been performed with transient good, but it is like the operation of replanting teeth; it will not do to tie to. The tyro at this business will feel certain that he has made a successful operation, but in time his house will come tumbling about his ears. The fangs of his replanted tooth will be eaten up as grubs eat a succulent root, and his remnant of a pulp will object to living in its decapitated state. We are told that nerves in various parts of the body are severed and afterward reunite. So they do, undoubtedly, but when this fact is instanced to show with what impunity the tooth pulps may be handled, we object to the illustration. The nerve and vessels confined within a tooth, and receiving nutrition through the minute opening at the apex, are in a far different condition from those traversing tissues and receiving health from every direction. The protection of pulps that have lost part of their substance will, I believe, ever be but an experiment, with the odds against such success as ought to justify the practice. In all these cases I believe the proper practice to be extirpation by the most approved methods. We often resort to capping when reason and experience say extirpate, solely because of the pain often necessary in nerve destruction. Many times little pain is occasioned, but generally, as you are aware, "a howling pain," to quote another, sends the patient back to you. Sedatives may be useful in allaying the irritation, but this requires time which is not always at our disposal. Before disease has progressed so far as to necessitate extirpation, we have exposed pulps which experience and history tell us can almost invariably be made to continue their normal functions. These exposures may possibly have caused pain through thermal changes or other irritations, of transient character, passing away and leaving the integrity of the pulp undisturbed. In such cases we have a possible chance for success. The first difficulty is in ascertaining the exact condition. The history of the case is often misleading, and the best plan is, after drying the cavity, to insert a non-irritant stopping, and after it shall have remained for a suitable time, to remove it for careful examination. Any heroic means must be avoided, for if the pulp is free from congestion, any violent handling, such as puncturing or lacerating, is prejudicial to success.

The process of carefully disinfecting must come next, and whether performed with carbolized water, eucalyptus oil, salicylic acid, listerine, alcohol, or any of the other standard remedies, it should be done with those of such strength and temperature as to be grateful to the sensitive pulp. The common practice of using ice-cold applications is hurtful, cruel, and unnecessary. The medicine, with a little warming, will penetrate better and be more active. After disinfecting and thoroughly drying, there seems to me nothing superior to fine silk adhesive plaster, or sandarach varnish, for the first covering of the pulp. After drying, which is speedily accomplished, another piece of plaster or coating of varnish can be given, until thorough protection is afforded. Upon this can be introduced the foundation filling of cement, as usual. This capping is introduced without the slightest pressure upon the pulp; it hermetically seals the exposure, and seems to be a perfect protection without irritation. If the sandarach covering is first used, it may be reinforced with a layer of the silk adhesive plaster before introducing the cement filling for foundation.

If pulp exposure has gone a step further, to the verge of suppuration, the exposure may first be covered with a thick paste of the oxide of zinc and creosote. In all cases, if gold is to be used, it should be inserted at another sitting. There is no safety in finishing at the same operation, on account of liability of disturbing the not yet hardened cement.

If after-trouble ensues, that will not readily yield to treatment, the filling and capping must be removed. If the exposure and chances for success have been wrongly judged, the sooner a radical correction is made the better for patient and operator.

There is still another quite distinct class of cases, where decay has reached the pulp and yet no exposure has occurred. The inorganic components of the tooth are gone, dissolved by the acids, and the scavengers have not yet removed the organic part, which occupies its original position and saves the pulps from actual exposure. We often find pain has occurred here also, and sometimes the pulp is beyond help; but this is rare, and without actual exposure the operation of pulp protection results in certain success. Formerly the remaining covering was removed before proceeding to cap, but this suicidal practice has been abandoned, and every care is taken not to expose by removing the layer of the organic part of dentine, but to carefully disinfect and proceed as before stated. This natural capping, properly treated, will remain unchanged for an indefinite time.

My object in this essay has been to advise—

1st. The extirpation of the pulp when disease has progressed so as to destroy a portion of its substance, instead of endeavoring to preserve what remained.

2d. In capping, to use the non-irritating treatment.

3d. To especially recommend the silk adhesive plaster and sandarach varnish for first covering of the exposed or nearly exposed pulps.

In all, I have spoken from careful observation and experience; spoken guardedly because I know how fallible individual experience may be. The methods advised are not new, but yet they are not those generally practiced at the present day.

